# Comparison of Batch and Continuous Wet-Processing of Coffee: Changes in the Main Compounds in Beans, By-Products and Wastewater

**DOI:** 10.3390/foods9081135

**Published:** 2020-08-18

**Authors:** Gustavo A. Figueroa Campos, Sorel Tchewonpi Sagu, Pedro Saravia Celis, Harshadrai M. Rawel

**Affiliations:** 1 Institute of Nutritional Science, University of Potsdam, Arthur-Scheunert-Allee 114-116, 14558 Nuthetal, Germany; figueroacamp@uni-potsdam.de (G.A.F.C.); sorelsagu@uni-potsdam.de (S.T.S.); 2 Regional School of Sanitary Engineering and Water Resources (ERIS), University of San Carlos of Guatemala, Ciudad Universitaria Zona 12, Guatemala 01012, Guatemala; pcsaraviac@gmail.com

**Keywords:** Arabica coffee beans, coffee by-products, batch process, continuous process, nutritional characteristics

## Abstract

Many technical challenges still need to be overcome to improve the quality of the green coffee beans. In this work, the wet Arabica coffee processing in batch and continuous modus were investigated. Coffee beans samples as well as by-products and wastewaters collected at different production steps were analyzed in terms of their content in total phenols, antioxidant capacity, caffeine content, organic acids, reducing sugars, free amino group and protein content. The results showed that 40% of caffeine was removed with pulp. Green coffee beans showed highest concentration of organic acids and sucrose (4.96 ± 0.25 and 5.07 ± 0.39 g/100 g DW for the batch and continuous processing). Batch green coffee beans contained higher amount of phenols. 5-caffeoylquinic Acid (5-CQA) was the main constituent (67.1 and 66.0% for the batch and continuous processing, respectively). Protein content was 15 and 13% in the green coffee bean in batch and continuous processing, respectively. A decrease of 50 to 64% for free amino groups during processing was observed resulting in final amounts of 0.8 to 1.4% in the processed beans. Finally, the batch processing still revealed by-products and wastewater with high nutrient content encouraging a better concept for valorization.

## 1. Introduction

Coffee is one of the most widely produced and traded agricultural products. World production in 2019 was estimated at around 167 million 60-kg bags, with 95 million bags of Arabica (*Coffea arabica*) and 72 million bags of Robusta (*Coffea canephora*) [1]. Robusta coffee has a stronger and harsher taste with a high concentration of caffeine, while Arabica coffee, considered to be of higher quality, has a sweeter taste, with a higher amount of sugars and an excellent acidity evaluation [2]. Besides Arabica and Robusta coffees, there are species such as Libercia (*Coffea liberica*) and Excelsa representing around 1% of world production, mainly grown in Africa and of low commercial importance [3]. Green coffee beans are rich in bioactive compounds such as caffeine, free amino groups, polyphenols and chlorogenic acids. They contain a number of functional biological properties such as stimulation of the central nervous system, myocardial stimulation and peripheral vasoconstriction [4]. These characteristics of green coffee beans have been subject of numerous works and interest from the nutraceutical and pharmaceutical industries [5]. Although taking into account these different classifications, the composition and concentration of coffee constituents differ according to the type of coffee beans, the environmental conditions, soil type and geographical areas of cultivation [6,7].

Two methods are generally used to process coffee cherries: the dry method, also known as the natural method, and the wet also called the washing method. Processing of coffee cherries by dry or wet method is principally similar for all varieties and generally involves elimination of the five layers (skin, pulp, parchment, mucilage and silver skin) in order to release the coffee beans, also defined as green coffee beans. The dry method consists of drying the coffee cherry just after harvest and is followed by mechanically de-husking. Cherries are later cured and the product obtained is termed in trading society as cherry coffee [8]. It is preferably the simplest way of processing coffee cherries, but also represents a laborious method for obtaining high quality coffee beans [9].

The wet-processing method has become more common and is used by many coffee producers to meet market demands for green coffee beans with higher quality. The wet process also ensures optimal protection against oxidation and top quality. Large amounts of clean water are, however, required (40 L/kg of dry parchment coffee) [10]. In the wet method, the layers are successively removed in a succession of steps including pulping, fermentation, washing and drying [11]. Pulping consists of removing the red outer skin and the white fleshy pulp. Fermentation aims to degrade the mucilage, which consists of hemicelluloses, pectin substances and sugars [12]. During fermentation, natural enzymes and bacteria operate together to break down the mucilage (the amorphous and water-insoluble gel). Beans are washed, dried to a moisture content of about 10–12%, and the thin layers of parchment around the coffee beans are then removed using a de-husker [13]. This method allows during the fermentation step the release of reducing sugars and free amino acids. Green coffee beans are later roasted, constituting the initial step in the preparation of beverages from coffee. Numerous and complex chemical reactions take place during roasting and one of the most important categories of these interactions is the Maillard reactions involving the participation of free amino acid and reducing sugars, finally contributing to the development of the color, aroma and taste of the roasted coffee beans has been discussed [8].

It has often discussed that processing factors were responsible for the specific flavor expressions of differently processed coffees [9]. In other words, the type of processing influences the differences in the constituents of green coffee which, during roasting, give rise to aroma compounds and express the characteristics of roasted coffee beans [14]. In addition, large amounts of co-products such as coffee pulp, mucilage and parchment, and wastewater are generated during the wet processing [15]. Skin and pulp represent about 43% *w*/*w*, mucilage and soluble sugars 11.8% *w*/*w*, and finally parchment about 6.1% *w*/*w* [16]. These characteristics also differ according to the mode of production and can be valorized in selected sub-sectors to a limited extent. If wet processing of coffee beans is being performed on a large scale, the untreated effluents produced will greatly exceed the self-purification capacity of the natural waterways. In order to overcome the pollution potential of processing such waste waters, a clear understanding of its constitution is necessary to design a feasible treatment system [17]. Further, studying on the effects of wet processing methods on the characteristics and quality of green coffee beans will also provide a broad range of improvement possibilities [18]. No studies of the effect of batch or continuous wet production on the characteristics of green coffee beans and by-products are currently available. It is in this context that the present work has been undertaken with the intention to compare the effects of the continuous and the batch wet processing, encompass the state-of-the-art methods and at the same time visualize the existing opportunities for further valorization. Samples from a batch and a continuous Arabica coffee production plant were collected at each stage of processing and analyzed for their total phenol content, antioxidant capacity, caffeine content and as well as monitoring the composition of organic acids, reducing sugars, free amino groups and protein content.

## 2. Materials and Methods

### 2.1. Materials

Samples from Arabica species were used in this work. Depulped coffee beans, initial fermented coffee beans, final fermented coffee beans, green coffee beans, coffee pulp, parchment, pulping coffee wastewater, fermentation coffee wastewater, and washing coffee wastewater were obtained from a pilot batch processing (Rio Colorado Company, located in Palencia, Guatemala) and from a continuous pilot process (Santa Sofia Company, located in Santa Rosa, Guatemala). Coffee bean and by-product samples were collected directly at each stage of the production, stored at −20 °C and then transferred to the laboratory under refrigeration conditions.

Ascorbic acid (Carl Roth GmbH, Karlsruhe, Germany) was used as standards for the determination of the antioxidant capacity. Chlorogenic acid (Sigma Aldrich, Steinheim, Germany) and gallic acid (Carl Roth GmbH, Karlsruhe, Germany) were used for the determination of phenolic compounds. All the other chemicals and reagents used were at least of analytical grade.

### 2.2. Methods

#### 2.2.1. Batch Pilot and Continuous Pilot Configurations for Coffee Cherries Processing

**Figure 1:**
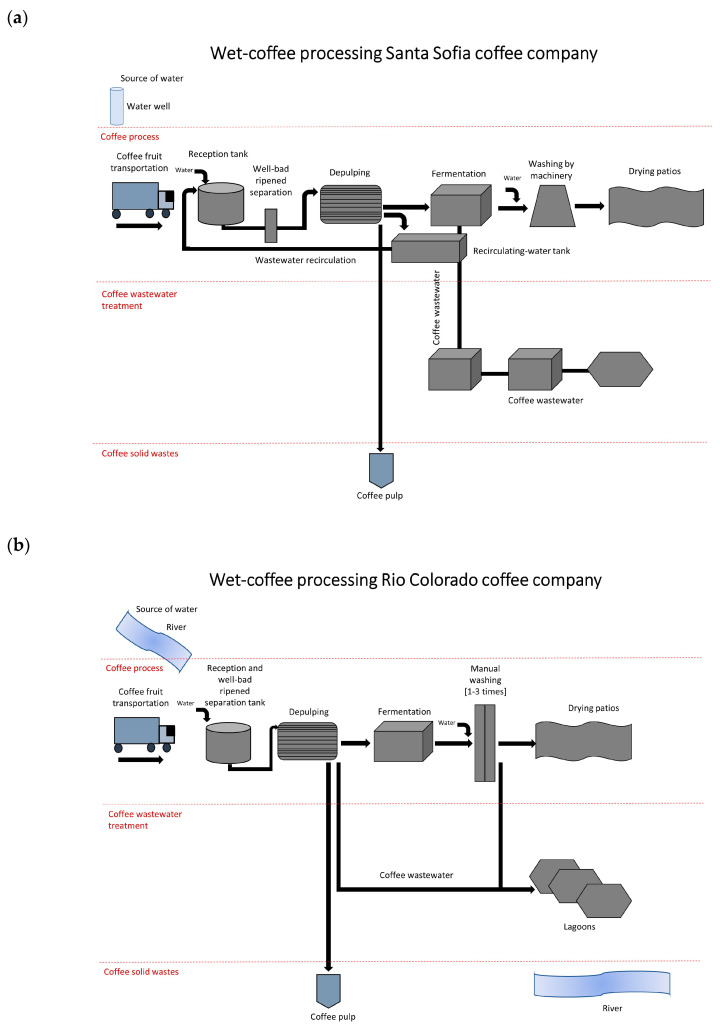
Batch (**a**) and continuous (**b**) wet-coffee processing.

Two major processing options (pilot batch and continuous wet processing, Figure 1) are commonly practiced in many wet coffee producing countries of Latin America and especially in Guatemala. They differ in the composition of the wash water used to produce the final dried coffee beans. Our observation, while visiting different small-scale companies in Guatemala, also indicated that slight individual changes to these two principle processing outlines may be employed. An overview of the different technical possibilities is given in a textbook on coffee production, Rothfos (1980) [19]. In the batch processing, the coffee cherries were immersed in water to select cherries between unripe and the good ripe ones. Cherry skin and pulp were removed by pressing the fruit through a sieving screen, and beans were put in a fermentation tank with a water stream for 26 h. Fermented coffee beans were washed out to remove mucilage and then finally sundried. Water applied for washing the fermented beans consisted of 50% fresh water and 50% wastewater coming from former (preceding) de-pulping and fermentation steps. This water was gathered and pumped back to the reception tank to be included in processing the next coffee batch. To process 60,000 kg of coffee cherries, a flow rate of 6.9 L/s for 1.5 h was applied. Therefore, 1 kg of processed coffee required ca. 0.63 L of water. Fresh water was used during the whole continuous processing and it required 48 hours to complete fermentation. Fermented coffee beans thereafter were allowed to rest in a shallow tank for another 40 h, followed by a washing step. To process 12,000 kg of coffee, a flow rate of 4.9 L/s for 2 h was generally applied, thus consuming at least 3 L of water, which was required to produce 1 kg of processed coffee. In comparison, a mechanical system was reported in Central America to remove the mucilage from the beans. This ecological process required only ca.1 L/kg of dry parchment coffee and largely preserved the original quality of the coffee beans [10].

#### 2.2.2. Sample Preparation

Sample preparation for analyses was conducted according to the type of sample (bean and solid by-products or wastewater samples). Coffee beans, pulp and parchment samples were freeze-dried (Martin Christ GmbH, Osterode am Harz, Germany), grounded with a laboratory blender (Moulinex, Écully, France) and then fractionated to obtain flours with particle sizes <0.2 μm.

To determine the total phenolic content, antioxidant capacity, reducing sugars and free amino groups, 10 mg of flour was mixed with 1 mL of 80% methanol in water (80:20, *v*/*v*). Extraction was performed at room temperature under shaking conditions for 30 min. After centrifugation at 9300× *g* for 10 min, supernatants were collected and stored at −20 °C. Likewise, wastewater samples were directly centrifuged, supernatants collected and stored at −20 °C.

To analyze the organic acids, extraction was performed under the same conditions as described above, using distilled water as solvent. Wastewater samples were directly centrifuged. The supernatants were collected and applied for solid phase extraction (SPE) using a column containing 300 mg of MN SC 6 Polyamide. After activating the column with 3 mL of 0.01 N sulfuric acid, 1 mL of sample was loaded, washed/eluted with 0.01 N sulfuric acid, and stored at −20 °C. Thereafter, the organic acids remained in the washing out phase. The treatment also allows the removal of different compounds (e.g., colored fractions) which may interfere with the analysis of the organic acids.

To determine the protein contents, 10 mg of flour was initially mixed with 1 mL of 80% methanol for 30 min and centrifuged (9300× *g*, 10 min at 4 °C). Supernatants were removed, the precipitates washed with 1 mL of acetone for 5 min and dried under a hood. Extraction was done overnight using 1 mL of 1% sodium dodecyl sulfate containing 1 mM dithioerythritol. Mixtures were then centrifuged (9300× *g*, 5 min at 4 °C), the supernatants collected and stored at −20 °C.

#### 2.2.3. Folin–Ciocalteu Method for the Analysis of Total Phenolic Content

The total phenolic content was determined using the Folin–Ciocalteu method as described by Singleton, et al. [20] with some modifications. Gallic acid was used as standard. Briefly, 20 µL of sample or standard solutions was mixed with 180 µL of the Folin reagent (mixture of Folin–Ciocalteu and 0.01 M NaOH, 1:1, *v*/*v*) on the iMark microplates while using the corresponding reader (Bio-Rad Laboratories, Hercules, CA, USA). The mixtures were incubated at room temperature for 30 min and absorbance was measured at 750 nm. The results were expressed as g/100 g gallic acid equivalent (GAE) of dry weight (DW) for coffee beans and by-product samples; and as mg/L GAE for wastewater samples.

#### 2.2.4. Determination of Caffeine Content and Composition of Major Phenolic Compounds by High-Performance Liquid Chromatography (HPLC)

Caffeine content and composition in phenolic compounds were determined using a HPLC system (Shimadzu HPLC system GmbH, Leonberg, Germany). Analyses were performed with a C18 column (150 mm × 4.6 mm, Ø 3 µm, at 40 °C; BISCHOFF GmbH, Leonberg, Germany) with a pore size of 120 Å. Flour samples were mixed in a ratio of 10:1 (*w*/*v*) with a solution of 50% methanol containing 1% acetic acid. Wastewater samples were mixed in a ratio of 1:1 (*v*/*v*) with a solution of 98% methanol containing 2% of acetic acid. An amount of 10 µL of each solution was injected onto the column. The separation was performed at a flow rate of 1 mL/min for 45 min. Mobile phases consisted of 0.1% trifluoroacetic acid in distilled water (eluent A) and methanol (eluent B). The gradient applied was 20% eluent B from 0.01 to 3 min; 35% eluent B, from 3 to 20 min; 68% eluent B from 20 to 37 min; 68% eluent B from 37 to 40 min; 20% eluent B from 40 to 45 min. The detection was performed with a dual wavelength; at 280 nm for the determination of caffeine and at 325 nm for the determination of phenolic compounds (with an UV-Vis SPD-10 AVP detector, Shimadzu, Kyoto, Japan). The results were expressed in g/100 g chlorogenic acid equivalent (CAE) DW for coffee beans and by-product samples and as mg/L CAE for wastewater samples.

#### 2.2.5. Reducing Sugars

Reducing sugars were determined under isocratic conditions with a Shimadzu HPLC system equipped with an evaporative light-scattering detection (Shimadzu ELSD-LT II, Gain = 8, Shimadzu, Kyoto, Japan). Samples were analyzed on an X-bridge Amide column (100 mm × 4.6 mm with particle size of 3.5 µm; Waters GmbH, Eschborn, Germany). Column and ELSD temperatures were set at 65 °C and 40 °C, respectively. The mobile phases consisted of solutions of 84% acetonitrile and distilled water (eluent A) and 0.1% ammonia (eluent B). Flow rate was fixed at 0.8 mL/min for an injection volume of 5 µL. Fructose, glucose, sucrose, and maltose solutions prepared with 60% methanol were used as external standards for a calibration range from 0.00 to 0.4 mg/mL. The results were expressed as g/100 g DW for coffee beans and by-product samples and as mg/L for wastewater samples.

#### 2.2.6. Determination of Organic Acids

HPLC system (Shimadzu Europa GmbH, Duisburg, Germany) equipped with an UV-Vis Detector SPD-10 AVP (Shimadzu, Kyoto, Japan) was used to analyze the organic acids. A volume of 50 µL of samples was injected into an Aminex HPX-87H column (300 mm × 7.8 mm, Ø 9 µm, 65 °C). The separation was done under isocratic conditions with solution of 0.01 N sulfuric acid. Flow rate of 1.0 mL/min was applied for a separation time of 20 min. Citric acid, malic acid, lactic acid, acetic acid, propionic acid and butyric acid were used as standards for a calibration range from 0.00 to 0.2 mg/mL. The detection was completed at 210 nm and the final results were expressed as g/100 g DW for coffee beans and by-product samples and as mg/L for wastewater samples.

#### 2.2.7. Antioxidant Capacity

Antioxidant capacity was measured with the Ferric Reducing Ability of Plasma (FRAP) method using ascorbic acid as standard. FRAP reagent was prepared by mixing in a ratio of 1:1 20 mM of iron (III) chloride hexahydrate with 10 mM of 2,4,6-Tripyridyl-*S*-Triazine (TPTZ). Samples or standard solutions (10 µl) were then mixed with 150 µL of FRAP reagent and then incubated at room temperature for 6 min. The absorbance was taken at 595 nm using the microplate absorbance reader (Bio-Rad Laboratories, Hercules, CA, USA). The results were expressed as g/100 g FRAP equivalent (FE) of dry weight (DW) for coffee beans and by-product samples and as mg/L FE for wastewater samples.

#### 2.2.8. Proteins and Free Amino Groups

Protein content was measured by using the method of Lowry, et al. [21] with BSA as standard. Free amino groups were analyzed using the fluorescamine free amino groups assay. Fluorescamine reagent was prepared by mixing 3 mg of fluorescamine (Thermo Fischer Scientific, Waltham, MA, USA) with 1 mL of dimethyl sulfoxide (DMSO). Samples or standard solutions (150 µL) were mixed with 50 µL of fluorescamine reagent and incubated at room temperature for 15 min. Excitation was set at 365 nm and emission at 470 nm while using a microplate reader (TECAN infinite M200 PRO, Männedorf, Switzerland). l-Leucin with concentrations ranging from 0.00 to 6.00 mM was used as standard and the results were expressed as g/100 g DW for coffee beans and by-product samples and as mg/L for wastewater samples.

#### 2.2.9. Sodium Dodecyl Sulfate-Polyacrylamide Gel Electrophoresis (SDS-PAGE)

SDS-PAGE was performed under reducing conditions using Novex™ NuPAGE™ 4–12% Bis-Tris gels according to the manufacturer’s instructions (Thermo Scientific™, Carlsbad, CA, USA). Samples were mixed with Novex™ NuPAGE™ LDS sample buffer to a ratio of 1:1 and then heated a 95 °C for 10 min. An amount of 10 μL of the mixture was loaded into the gels. After separation, gels were stained overnight with a solution of Coomassie blue R250, then destained for ca. 2 h with 10% acetic acid and finally scanned (Bio-5000 Professional VIS Gel Scanner, SERVA Electrophoresis GmbH, Heidelberg, Germany). Spectra Multicolor Broad Range Protein-Marker (Thermo Fisher Scientific, Vilnius, Lithuania) was used as standard.

#### 2.2.10. Data Analysis

All experiments were carried out at least in triplicate and data were expressed as mean ± standard deviation. Data were analyzed with GraphPad Prism 8^®^ (GraphPad Software, Inc., San Diego, CA, USA) using two-way ANOVA and Tukey’s test, and the results were considered statistically significant for *p* < 0.05.

## 3. Results and Discussion

### 3.1. Caffeine, Total Phenols and Antioxidants Analysis

**Figure 2:**
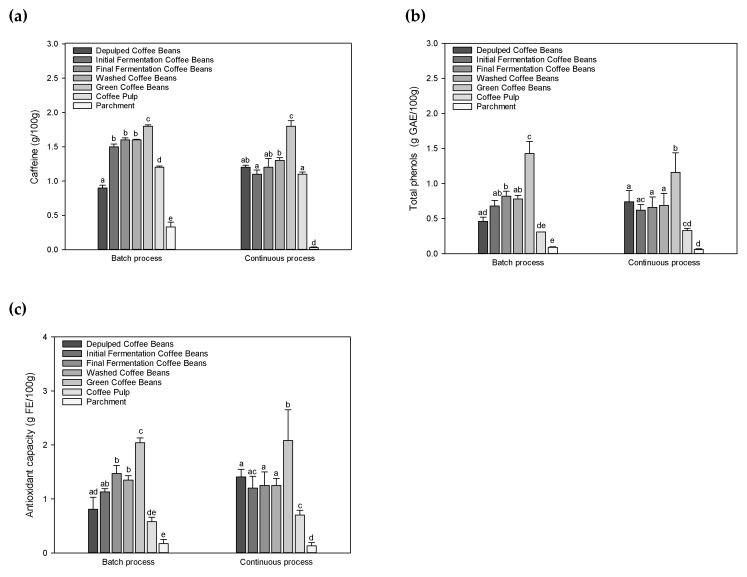
Content of (**a**) caffeine, (**b**) total phenols and (**c**) antioxidant capacity of coffee beans and by-products during the wet batch and continuous processing. The antioxidant capacity is in Ferric Reducing Ability of Plasma (FRAP) equivalents (FE). Data are expressed as means ± standard deviation *n* = 3. Different letters indicate significantly different values for each group (different superscript letters indicate a significant different, *p* < 0.05, ANOVA, Tukey’s test).

Coffee beans, coffee pulp and parchment were collected at different steps of the wet batch and continuous pilot scale processing, and samples were analyzed. Figure 2 presents the concentrations of caffeine, phenolic compounds and antioxidant capacity of de-pulped coffee beans, initial and final fermented coffee beans, washed coffee beans, green dried coffee beans samples, coffee pulp and parchment. An increase in caffeine, total phenols and antioxidant capacity from the de-pulped coffee beans to the green coffee beans can be observed, depending on whether the cherries were processed in batch or in continuous workflow. Caffeine content in de-pulped coffee beans and green coffee beans were 0.85 ± 0.04 and 1.21 ± 0.03; and 1.76 ± 0.02 and 1.78 ± 0.08 g/100 g DW for the batch and the continuous process, respectively. Statistical analysis shows that there were no significant differences while comparing the values in green coffee beans. Contents of caffeine in Arabica green coffee beans were reported to range between 0.7 and 1.7 g/100 g [22,23]. Caffeine values of coffee beans at different processing steps were all in that range. High values of caffeine were obtained in coffee pulp (1.16 ± 0.02 and 1.09 ± 0.03 g/100 g DW for the batch and continuous processing, respectively), and caffeine was detected in the parchment of the batch process (Figure 2a). This result indicates that up to 40% of caffeine is removed with pulp during the wet processing, regardless of whether the processing is performed in batch or in a continuous way. Caffeine (1,3,7-trimethyl-xanthine) was reported to be basically related to the protective function of coffee beans and viewing the inner of the cherry, caffeine is found as much in the beans as in the pulp and skin [24]; justifying the higher amount of caffeine in the pulp documented here.

Phenolic compounds and antioxidant capacity were analyzed, and data processing did not show a significant difference in green coffee bean samples (*p* < 0.05). The values of 1.43 ± 0.18 and 1.16 ± 0.28 g/100 g DW; 2.04 ± 0.09 and 2.08 ± 0.57 g/100 g DW were obtained for the batch and the continuous processing, respectively (Figure 2b,c). Significant differences in terms of antioxidant capacity were observed between the de-pulped coffee beans (0.81 ± 0.22 and 1.41 ± 0.14 g FE/100 g for the batch and the continuous processing, respectively).

Moreover, significant changes in terms of total phenol and antioxidant capacity were recorded during the fermentation step in the batch processing (*p* > 0.05) while they remained constant in the case of the continuous process. Total phenol values of 0.68 ± 0.08 and 0.82 ± 0.07 g GAE/100 g DW; and antioxidant capacity values of 1.13 ± 0.06 and 1.47 ± 0.15 g FE/100 g DW were obtained for the initial and the final fermentation coffee beans with the batch processing, respectively. These values were 0.62 ± 0.08; 0.66 ± 0.15; 1.20 ± 0.22 and 1.25 ± 0.25 g/100 g DW for the total phenol and antioxidant capacity for initial and final step of fermentation of coffee beans during the continuous processing, respectively. Similar values of phenolic content and antioxidant capacity were previously reported from Arabica green coffee beans [22,23,25].

**Figure 3:**
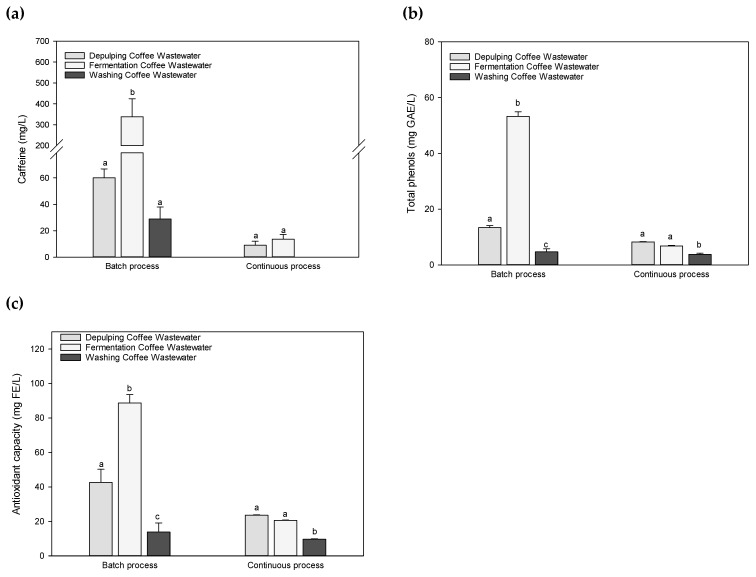
Amounts of (**a**) caffeine content, (**b**) total phenols and (**c**) antioxidant capacity of de-pulping coffee wastewater, fermentation coffee wastewater and washing coffee wastewater during the wet batch and continuous processing. Data are expressed as means ± standard deviation. *n* = 3. Different letters indicate significantly different values for each group (different superscript letters indicate a significant different, *p* < 0.05, ANOVA, Tukey’s test).

Wastewater resulting from the de-pulping, fermentation and washing steps were also investigated and the results are presented in the Figure 3. It can be seen that batch processing exhibited higher contents than continuous processing. Values of caffeine, total phenols and antioxidant capacity of wastewater from the fermentation step in batch processing were 388.6 ± 85.9; 53.2 ± 1.6 and 88.7 ± 4.9 mg/L, respectively. Corresponding values for the continuous processing were significantly different (*p* < 0.05):13.6 ± 3.6; 6.8 ± 0.2 and 20.5 ± 0.2 mg/L, respectively. The results document an increase in caffeine content up to five times in the wastewater resulting from the fermentation step of the batch processing. During the fermentation, organic acids are also produced and it has been shown that caffeine solubility may increase with acidity of the medium [24]. The main reason for the observed increase in caffeine but also that of phenolic compounds and the corresponding antioxidant capacity most probably lies in the re-using of the wastewater from former/proceeding treatments during the batch processing. Caffeine was not detectable in wastewater generated by the washing step in the continuous processing and the corresponding total phenols and the antioxidant capacity values were not significant (*p* < 0.05) with 3.7 ± 0.5 and 9.6 ± 0.3 mg/L as compared to batch processing, respectively. These results clearly show that the composition of wastewater is dependent on how much and how often the wastewater is recycled in the batch processing. Thereafter, the first indication for the potential of valorization is given in the proper re-utilization for the so-produced wastewater. Wastewater after being used is disposed in lagoons where one part is evaporated and the other one is infiltrated into the subsoil. The effect of the nutrient density in the recycled wastewater, especially of phenolic compounds, may thereafter not only influence the micro-organism development but eventually also their composition during the fermentation; studies in this respect have not yet been conducted.

**Figure 4:**
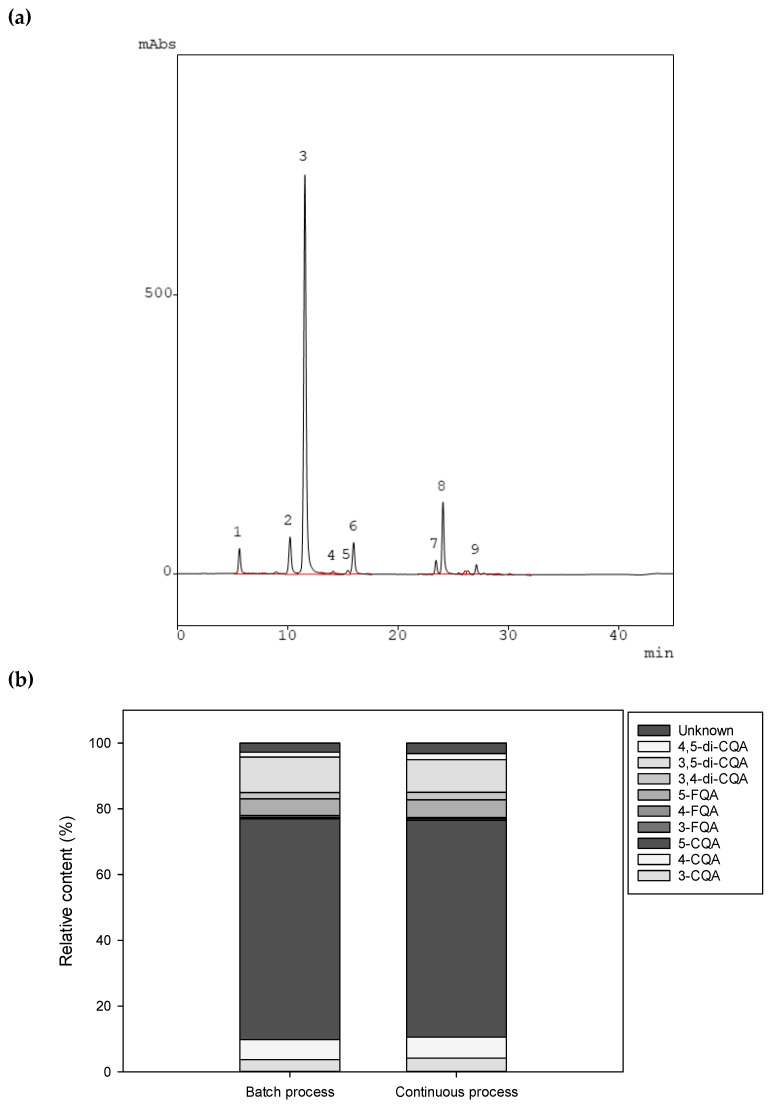
An exemplary chromatogram of green coffee beans obtained from batch processing (**a**) and the composition of the major phenolic compounds (**b**). (1) 3-*O*-Caffeoylquinic acid (3-CQA), (2) 4-*O*-Caffeoylquinic acid (4-CQA), (3) 5-*O*-Caffeoylquinic acid (5-CQA), (4) 3-*O*-Feruloyquinic acid (3-FQA), (5) 4-*O*-Feruloyquinic acid (4-FQA), (6) 4-*O*-Feruloyquinic acid (5-FQA), (7) 3,4-*O*-Dicaffeoylquinic acid (3,4-di-CQA), (8) 3,5-*O*-Dicaffeoylquinic acid (3,5-di-CQA), (9) 4,5-*O*-Dicaffeoylquinic acid (4,5-di-CQA), allocation as reported in [26].

The composition of green coffee beans for the major chlorogenic acids was analyzed using the HPLC and a total of 9 caffeoylquinic acid (CQA) and feruloylquinic acid (FQA) isomers were identified as described in our former work [26]. There were similarities in the composition for the green coffee beans resulting from both processing options. Main constituents were caffeoylquinic and feruloylquinic acid esters 5-CQA (67.1% and 66.0%), 4-CQA (6.1% and 6.4%), and 5-FQA (5.1% and 5.4%); and the dimer form 3,5-di-CQA (10.8% and 10.0%) for the batch and the continuous processing, respectively (Figure 4). Chlorogenic acids found in coffee beans are valued for their health benefits. They are known to have antimicrobial activity, preventing the degradation of bioactive compounds and strongly influencing the taste and color of coffee beverages [27]. The presented results clearly show that carrying out the wet-processing in batch or in continuous way does not change the pattern of the major phenolic compounds present. The proportion and especially the composition of these compounds remains more or less constant, but the overall total content is likely to be effected as documented in Figure 1.

### 3.2. Organic Acids

Organic acids, especially in wet coffee processing, are constituents of interest due to their different functionalities. In order to guarantee the proper drying of green coffee, mucilage layer needs to be degraded and this process takes place during the fermentation stage. It was showed that mucilage layer degradation was correlated to acidification by lactic acid bacteria [28,29,30,31]. Digested mucilage being precipitated out of the solution, making a thick crust on the surface of wastewater [2]. Results of organic acids analysis are compiled in Table 1. Data indicate that citric acid, malic acid and lactic acid were found to be present in the coffee bean samples and by-products during the different steps of treatment, either in the batch or the continuous processing. As it can be seen, among the coffee beans, green coffee beans showed the highest concentration of organic acids. Citric acid was found to be the predominant organic acid. Concentrations ranged from 0.6 to 1.0 g/100 g DW and from 0.7 to 1.0 g/100 g DW in the batch and the continuous system, respectively. Concentrations were higher in coffee pulp than coffee beans. For example, malic acid was found to be six times higher in pulp than green coffee beans when operating in continuous system, and about four times with batch processing (Table 1). Coffee pulp exhibited the highest concentrations in malic acid, 1.6 ± 0.05 g/100 g for batch processing and 3.4 ± 0.26 g/100 g for continuous processing. Fermentation is mainly carried out by *lactic acid bacteria*, *enterobacteriaceae*, and *bacillus* [12]. They are heterofermentative producing acetic and lactic acids [31]. However, malic and citric acid confer desirable acidity to the coffee beverage [32,33,34,35]. Moreover, enzymes produced from lactic acid fermentation might lead to the hydrolysis of macromolecules such as proteins, carbohydrates and polyphenols generating aroma precursors assuming that they can penetrate e.g., by diffusion into the coffee beans [36].

It can be clearly seen that for batch processing, lactic acid (2687.1 ± 23.0 mg/L) and propionic acid (1679.3 ± 67.9 mg/L) were the main organic acids in coffee wastewater. With the continuous processing, a concentration of 373.5 ± 15.0 mg/L of lactic acid in fermentation wastewater was determined. Propionic acid was not detected. Production of butyric and propionic acids during the fermentation indicate an over-fermentation, which can be responsible for the “stinker oniony” coffee aroma profiles [31]. This observation is supported by the fact that, during the fermentation process, microorganisms use part of nutrients from coffee beans to support their growth while producing secondary metabolites [36]. As the water is only partly renewed in the case of the batch process, it results in an accumulation of secondary metabolites, especially organic acids. There is little information about the effect of water recirculation on coffee bean constituents. Vásquez Morera [37] showed that acidity and aroma of coffee beans increased when recirculating water for two days of fermentation. Contact time between water and coffee beans might also increase acidity and decrease bitterness [38]. The enzymes and organic acids produced from fungal and bacterial fermentation lead to the hydrolysis of macromolecules such as carbohydrates, proteins and polyphenols resulting in simplified products like reducing sugars, amino acids and organic acids and these are important aroma precursors in roasting process which will impact the coffee beverage final cup quality [39,40].

Temperature and pH of wastewater along both processes were measured using a portable multiparameter HI98194 (HANNA instruments, Woonsocket, RI, USA). For batch processing, de-pulping water showed 17.8 °C and pH 4.5; fermentation water 18.2 °C and pH 4.2; and washing water 16.8 °C and 4.78 pH. With continuous processing, de-pulping water had 16.2 °C and pH 6.3; fermentation water 15.8 °C and pH 6.4; and washing water 13.7 °C and pH 7.4. These results were obtained via field measurements and document that the conditions of fermentation appear to be different. At the same time these slight differences during the treatments may also influence the degradation/biochemical interactions occurring during the processing and eventually also affect the quality of the coffee beans produced.

### 3.3. Reducing Sugars

Low molecular weight carbohydrates are involved in Maillard reactions and color changes during the roasting of green coffee beans. Table 2 illustrates the composition of reducing sugars of coffee beans and by-product samples during the wet batch and continuous processing. Five sugars were detected, including arabinose, fructose, mannose, glucose and sucrose. With the batch processing, arabinose contents in de-pulped coffee beans, coffee beans at the beginning of the fermentation, coffee beans after final fermentation, and coffee pulp, were 0.22 ± 0.01; 0.13 ± 0.04; 0.11 ± 0.01 and 0.18 ± 0.02 g/100 g DW, respectively. Arabinose as well as fructose and mannose were not detected in green coffee beans and values of glucose and sucrose were of 0.66 ± 0.04 and 4.96 ± 0.25 g/100 g DW, respectively. After the de-pulping step, there was no sucrose left in the coffee pulp. However, the concentrations of arabinose (0.18 ± 0.02 g/100 g DW), fructose (9.48 ± 0.43 g/100 g), mannose (0.92 ± 0.08) and glucose (8.72 ± 0.48 g/100 g) were significantly higher in coffee pulp. Although sucrose was not detected in parchment and wastewater samples, all the other reducing sugars (fructose, mannose and glucose) were present in a higher concentration in wastewater resulting from de-pulping, fermentation and washing. These results indicate removal of reducing sugars during the batch processing; sugars in wastewater are most likely liberated from coffee pulp and from mucilage layer which precipitate after fermentation.

In the case of the continuous processing, although arabinose was not detected in the coffee bean samples, fructose, mannose, glucose and sucrose were present at different concentrations. Fructose (9.74 ± 0.72 g/100 g DW), mannose (0.88 ± 0.08 g/100 g DW) and glucose (8.29 ± 0.59 g/100 g DW) in coffee pulp were similar to those obtained with the batch process. The analysis of the variance showed that differences were not significant. Although, other sugars like fructose and mannose may be present in green coffee beans, glucose and sucrose seem to be the most relevant ones independent of the processing conditions used. Values of 0.58 ± 0.05 and 5.07 ± 0.39 g/100 g DW were obtained for glucose and sucrose, respectively. Acidri, et al. [41] and Kinyua, et al. [42] showed that sucrose is the main low molecular weight saccharide component of the coffee seeds. Its higher content is correlated with a better cup quality. Depending on the type of coffee species, sucrose ranges from 5.1 to 9.4% in Arabica coffee have been documented [40].

From Table 2 higher glucose values were obtained in coffee pulp while those of sucrose were recorded in green coffee beans. We can see that with the batch processing glucose was significantly reduced between the de-pulping (4.91 ± 0.19 g/100 g DW) and final fermentation (1.13 ± 0.07 g/100 g DW) steps while sucrose concentration was increased from 0.49 ± 0.03 to 2.10 ± 0.11 g/100 g DW (*p* < 0.05). Conversely, with continuous production, glucose decreased from 1.56 ± 0.12 to 0.52 ± 0.04 g/100 g DW from the de-pulping to the fermentation steps, while sucrose fluctuated from 2.66 ± 0.17 to 2.29 ± 0.23 g/100 g DW. The mucilage acts as a sort of barrier, a protective outer layer to isolate the bean from the rest of the cherry, preventing internal decomposition by enzymatic and kinetic processes during fermentation [9]. In the fermentation process, microorganisms use simple sugars present in coffee beans as a carbon source. Therefore, reducing sugars like glucose and fructose decrease as they are consumed by microorganisms during the fermentation [43]. Compared to the batch process, reducing sugars in the continuous processing were not detected in the parchment and were up to 18 times lower in the fermentation wastewater. The batch processing due to recirculation/reuse of water thus results in by-products and wastewater that contain more reducing sugars—again a potential which should be valorized.

### 3.4. Soluble Proteins and Free Amino Groups

**Figure 5:**
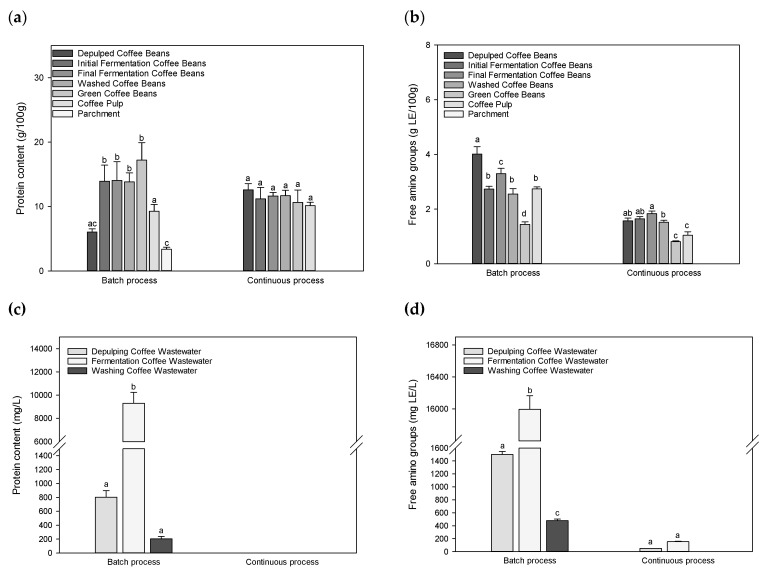
Amounts of protein content (**a**,**c**) and free amino groups (**b**,**d**) of coffee beans, by-products and wastewater samples during the wet batch and continuous processing. Data are expressed as means ± standard deviation. *n* = 3. Different letters indicate significantly different values for each group (different superscript letters indicate a significant different, *p* < 0.05, ANOVA, Tukey’s test).

Lowry method was used to quantify the soluble proteins in coffee beans and by-products. The results are presented in Figure 5. It appears that soluble proteins were present in considerable amounts in coffee samples, ranging from 5 to 15%. Protein content averages of 10–14% in coffee Arabica have been reported [44]. Different trends in the protein contents were exhibited during the processing. With the batch processing, an insignificant increase in protein contents during the fermentation step was registered, rising from 13.93 ± 2.50 to 14.05 ± 2.91 g/100 g DW from the initial to the final fermented coffee beans. This amount decreased with the washing step to a value of 13.58 ± 1.64 g/100 g DW in the washed coffee beans. Finally, protein content increased up to 17.21 ± 2.71 g/100 g DW in the green coffee bean after removing the parchment. The situation was similar with the continuous processing. An insignificant increase in protein content was observed between the coffee beans at the beginning of fermentation (11.20 ± 1.77 g/100 g DW) and the final fermented coffee beans (11.63 ± 0.56 g/100 g DW). After washing and parchment removal, the protein content in green coffee beans was 10.62 ± 1.94 g/100 g DW. The results indicate that the proteins (or their structures) might be affected during both processing options and a positive effect of the fermentation on the solubility and accessibility of proteins seems to be probable, while regarding the steps of de-pulping to initial fermentation, especially while considering batch process—further experiments are being conducted to address and substantiate these observations.

By-products were also analyzed and protein values of 9.25 ± 1.08 and 10.13 ± 0.53 g/100 g DW in coffee pulps were recorded for the batch and the continuous systems, respectively. Furthermore, while a protein content of 3.36 ± 0.33 g/100 g DW in the parchment sample of the batch system was registered, no protein was detected in the parchment from the continuous system. Similar behaviors were observed with wastewater samples. With batch system, protein values of 801.25 ± 94.6, 9292.5 ± 941.0 and 203.0 ± 34.0 mg/L were obtained for the wastewater form de-pulping, fermentation and washing steps, respectively, while protein was not detected in wastewater samples of the continuous system. The nature of these proteins will be addressed in the forthcoming experiments.

**Figure 6:**
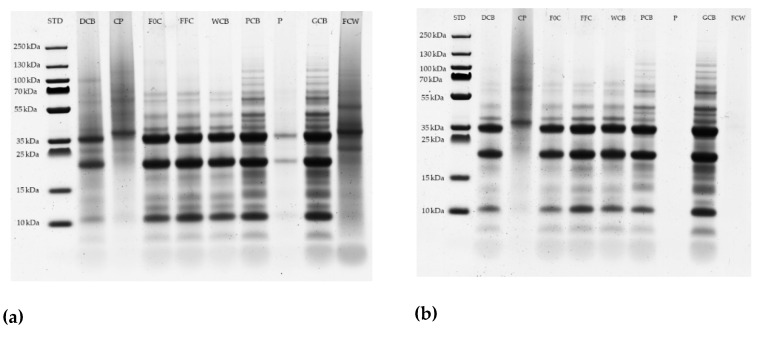
Electrophoresis of coffee beans, by-products and wastewater samples. (**a**) Batch and (**b**) continuous processing. With STD the protein ladder, DCB the de-pulped coffee beans, CP the coffee pulp, F0C the initial fermentation coffee beans, FFC the final fermentation coffee beans, WCB the washed coffee beans, PCB the parchment coffee beans, P the parchment, GCB the green coffee and FCW the fermented wastewater.

Storage protein accounts for 45% of total proteins in the green coffee beans. The most abundant of these proteins are the legumin-like seed storage proteins of the 11S size class [45,46,47]. The typical structure of an 11S storage protein consists of 3−6 monomers, which migrate into storage vacuoles (protein bodies) and generate by hydrophobic interactions the tri- and hexameric quaternary forms, with molecular weights of 150–400 kDa [48]. The rupture of the disulfide bonds in 11S monomers under reducing conditions releases the α (acidic) and β (basic) subunits [49]. *Coffea arabica* proteins, in absence of a reducing agent, showed subunit with molecular weight of 55 kDa, and in the presence of reducing agent (2-mercaptoethanol) consisted of two polypeptides with molecular weights of 33 and 24 kDa [45,47]. SDS PAGE was performed to check these main protein compositions. The scanned gels of samples from both processing options are presented in Figure 6. The Coomassie blue staining shows two main bands of 34 and 20 kDa protein in the de-pulped coffee beans. In both processing options, we observe an increase in the amount of higher molecular weight protein bands. These proteins could be a result from crosslinking of different smaller proteins or they are simply released better from the coffee bean matrix during the extraction. Further on-going experiments will encompass the nature of the composition of these protein bands. In the same context the nature of the main protein band(s) observed in the coffee pulp as well as in the fermentation wastewater (especially in the batch process) need also to be addressed. No bands were found in parchment from the continuous process, whereas parchment from the batch process shows a first band of about 36 kDa and a second band with a molecular weight of 22 kDa, and that corroborates with the results obtained from Lowry method where protein was detected in the parchment from batch process. The results of SDS-Page document some differences in the protein molecular composition. Further work planed here will show the nature of structural changes occurring to the individual proteins, especially in those of the major 11S storage protein along the processing steps.

Furthermore, in addition to the protein content, the fluorescamine assay was used to quantify amounts of free amino groups in coffee bean samples (free amino acid groups of proteins and peptides, and amino acids; Figure 5). For both processing configurations, the concentration of free amino groups was higher in the coffee pulp, while looking at the by-products of the processing. This shows that most of the protein remained intact in the coffee bean (see also results of SDS-PAGE, Figure 6), while the free amino groups were lost into the coffee pulp. With the batch process, statistical analysis showed a significant difference between the lowering values of samples from the final fermented, washed, and green coffee beans (*p* < 0.05). A lower amount of free amino groups (1.44 ± 0.09 g/100 g DW) was obtained in green coffee beans. Conversely, the free amino groups in the continuous processing remained more or less constant and the final significantly decreased value in green coffee bean (0.81 ± 0.03 g/100 g) was much lower than that obtained from the batch system (Figure 5b). This demonstrates that circulating fresh water during fermentation and washing operations contributed to a progressive leaching of free amino groups. From the de-pulped coffee beans to the green coffee beans, free amino groups decreased to about 64% in the batch processing and to about 50% in the continuous processing. Free amino groups in green coffee beans ranged between 0.8 and 1.4% in green coffee beans. In comparison, a free amino acid content in the range of 0.3–0.6% on a dry weight basis has been documented [10]. Considering the wastewater samples, free amino groups were more pronounced in batch processing samples compared to those of the continuous processing. The results showed that free amino groups increased in wastewater with fermentation. Fermentation wastewater values of 15,993.5 ± 170.9 and 153.5 ± 7.6 mg/L for the batch and continuous processing, respectively, were obtained. Values were about 100 times (keeping in mind that recirculation also increases the value documented) higher in batch processing comparing to the continuous processing. These data suggests that re-circulation primarily leads to increased values. If a significant degradation of proteins in coffee beans occurred during the fermentation step, especially when operating in batch, this cannot be interpreted from the observed values and still needs to be assessed more accurately. The results still do document a significant contribution originating from the processing from the de-pulped to green coffee beans, especially for the batch process. We can further assume that the increase may also result from the metabolic microbial turnover during the fermentation. This result reveals on one side the potential of reutilizing the wastewater with proper pre-treatment, but also gives the opportunity in changing the content of free amino groups in final green coffee bean. Knowing that this group of compounds is involved in the Maillard-Reaction during roasting, this possibility could be utilized to positively improve the coffee cup quality. Unfortunately, no detailed information is available to suggest that these free amino groups are either required or sufficient for the generation of the coffee aroma [10]. Further work is directed to encompass these aspects.

## 4. Conclusions

We hypothesized that the wet-coffee processing, whether in batch or continuous mode, can influence the physicochemical characteristics of green coffee beans, and thus later the roasting processing as well as the final cup quality. Thereafter, we found that the composition of green coffee beans from both batch and continuous production modes presented similarities. Further work will be directed towards the components that could participate in reactions taking part during the roasting. The by-products, especially during batch processing, revealed high concentrations of nutrients. The producers would profit in establishing appropriate simple technologies to re-use the pulp and wastewater for valorization.

## Figures and Tables

**Table 1:** Composition of organic acids in coffee beans, coffee by-products and wastewater during the batch and continuous processing.

		Coffee and By-Product Samples (g/100 g)							Wastewater Samples (mg/L)		
		DCB	F0C	FFC	WCB	GCB	CP	P	DCW	FCW	WCW
**Batch**	Citric acid	0.60 ± 0.03 ^a^	0.80 ± 0.05 ^b^	0.94 ± 0.01 ^c^	0.92 ± 0.02 ^cb^	1.03 ± 0.02 ^c^	1.25 ± 0.03 ^d^	0.16 ± 0.05 ^e^	72.0 ± 4.1 ^a^	847.0 ± 74.3 ^b^	68.8 ± 17.2 ^a^
	Malic acid	1.02 ± 0.00 ^a^	0.21 ± 0.00 ^b^	0.26 ± 0.06 ^b^	0.28 ± 0.07 ^b^	0.38 ± 0.02 ^b^	1.64 ± 0.05 ^c^	0.13 ± 0.02 ^b^	111.9 ± 3.0 ^a^	483.0 ± 26.8 ^b^	35.2 ± 0.7 ^c^
	Lactic acid	0.79 ± 0.07 ^a^	0.17 ± 0.04 ^b^	0.23 ± 0.01 ^b^	0.20 ± 0.00 ^b^	0.48 ± 0.03 ^c^	0.96 ± 0.00 ^d^	n.d	1082.1 ± 32.9 ^a^	2687.1 ± 23.0 ^b^	182.6 ± 7.2 ^c^
	Acetic acid	n.d	n.d	n.d	n.d	n.d	n.d	n.d	241.4 ± 26.6 ^a^	839.8 ± 12.0 ^b^	63.7 ± 8.8 ^c^
	Propionic acid	0.77 ± 0.23 ^a^	0.77 ± 0.23 ^a^	0.11 ± 0.01 ^b^	n.d	n.d	n.d	n.d	n.d	1679.3 ± 67.9	n.d
**Continuous**	Citric acid	0.74 ± 0.01 ^a^	0.87 ± 0.02 ^a^	0.69 ± 0.00 ^a^	0.71 ± 0.07 ^a^	0.99 ± 0.00 ^a^	1.20 ± 0.03 ^c^	n.d	n.d	8.0 ± 2.4	n.d
	Malic acid	0.63 ± 0.02 ^ab^	0.72 ± 0.00 ^a^	0.44 ± 0.01 ^b^	0.52 ± 0.09 ^ab^	0.56 ± 0.04 ^ab^	3.42 ± 0.26 ^c^	n.d	14.5 ± 0.2 ^a^	18.4 ± 0.6 ^a^	n.d
	Lactic acid	0.36 ± 0.01 ^a^	0.36 ± 0.02 ^a^	0.13 ± 0.02 ^b^	0.16 ± 0.01 ^b^	0.49 ± 0.05 ^c^	0.69 ± 0.08 ^d^	n.d	330.1 ± 8.4 ^a^	373.5 ± 15.0 ^a^	n.d
	Acetic acid	n.d	n.d	n.d	n.d	n.d	n.d	n.d	92.4 ± 3.3 ^a^	81.7 ± 2.5 ^a^	n.d
	Propionic acid	n.d	n.d	n.d	n.d	n.d	n.d	n.d	10.6 ± 3.3	n.d	n.d

Data are expressed as means ± standard deviation. *n* = 3; n.d = not detected. Different letters indicate significantly different values for each group (different superscript letters indicate a significant difference, *p* < 0.05, ANOVA, Tukey’s test). DCB: de-pulped coffee beans; F0C: initial fermentation coffee beans; FFC: final fermentation coffee beans; WCB: washed coffee beans; GCB: green coffee beans; CP: coffee pulp; P: parchment; DCW: de-pulping coffee waste water; FCW: fermentation coffee wastewater and WCW: washing wastewater.

**Table 2:** Composition of reducing sugars in coffee beans, coffee by-products and wastewater during the batch and continuous processing.

		Coffee and By-Product Samples (g/100 g DW)							Wastewater Samples (mg/L)		
		DCB	F0C	FFC	WCB	GCB	CP	P	DCW	FCW	WCW
**Batch**	Arabinose	0.22 ± 0.01 ^a^	0.13 ± 0.04 ^b^	0.11 ± 0.01 ^b^	n.d	n.d	0.18 ± 0.02 ^a^	n.d	25.0 ± 1.0 ^a^	610.3 ± 57.2 ^b^	24.7 ± 2.1 ^a^
	Fructose	5.63 ± 0.15 ^a^	0.59 ± 0.04 ^b^	0.66 ± 0.04 ^b^	0.39 ± 0.03 ^b^	n.d	9.48 ± 0.43 ^c^	n.d	43.3 ± 0.6 ^a^	327.7 ± 30.4 ^b^	47.0 ± 2.6 ^a^
	Mannose	0.70 ± 0.02 ^a^	0.21 ± 0.02 ^b^	0.18 ± 0.02 ^b^	0.16 ± 0.01 ^b^	n.d	0.92 ± 0.08 ^c^	0.17 ± 0.01 ^b^	1308.0 ± 46.6 ^a^	1720.3 ± 65.5 ^b^	151.0 ± 7.9 ^c^
	Glucose	4.91 ± 0.19 ^a^	0.91 ± 0.06 ^b^	1.13 ± 0.07 ^b^	0.80 ± 0.05 ^bd^	0.66 ± 0.04 ^bd^	8.72 ± 0.48 ^c^	0.23 ± 0.01 ^d^	253.3 ± 11.1 ^a^	1400.3 ± 88.6 ^b^	87.0 ± 5.6 ^c^
	Sucrose	0.49 ± 0.03 ^a^	1.69 ± 0.08 ^b^	2.10 ± 0.11 ^bc^	2.44 ± 0.17 ^c^	4.96 ± 0.25 ^d^	n.d	0.04 ± 0.01 ^e^	n.d	n.d	n.d
**Continuous**	Arabinose	n.d	n.d	n.d	n.d	n.d	n.d	n.d	34.7 ± 1.2 ^a^	34.7 ± 2.1 ^a^	n.d
	Fructose	1.68 ± 0.16 ^a^	1.81 ± 0.15 ^a^	0.20 ± 0.01 ^b^	0.27 ± 0.02 ^b^	n.d	9.74 ± 0.72 ^c^	n.d	n.d	29.0 ± 1.4	n.d
	Mannose	0.21 ± 0.01 ^a^	0.42 ± 0.04 ^b^	0.16 ± 0.00 ^a^	0.23 ± 0.02 ^a^	0.17 ± 0.01 ^a^	0.88 ± 0.08 ^c^	n.d	395.0 ± 14.2 ^a^	482.7 ± 22.2 ^b^	n.d
	Glucose	1.56 ± 0.12 ^a^	1.81 ± 0.15 ^a^	0.52 ± 0.04 ^b^	0.61 ± 0.04 ^b^	0.58 ± 0.05 ^b^	8.29 ± 0.59 ^c^	n.d	66.0 ± 3.6 ^a^	77.0 ± 2.6 ^a^	n.d
	Sucrose	2.66 ± 0.17 ^a^	2.05 ± 0.15 ^b^	2.29 ± 0.23 ^ab^	2.47 ± 0.18 ^a^	5.07 ± 0.39 ^c^	n.d	n.d	n.d	n.d	n.d

Data are expressed as means ± standard deviation. *n* = 3; n.d = not detected. Different letters indicate significantly different values for each group (different superscript letters indicate a significant different, *p* < 0.05, ANOVA, Tukey’s test). DCB: de-pulped coffee beans; F0C: initial fermentation coffee beans; FFC: final fermentation coffee beans; WCB: washed coffee beans; GCB: green coffee beans; CP: coffee pulp; P: parchment; DCW: de-pulping coffee waste water; FCW: fermentation coffee wastewater and WCW: washing wastewater.
